# Differences in treatment outcomes between ultrasound-guided percutaneous microwave ablation and endoscopic thyroidectomy for patients with papillary thyroid microcarcinoma: Protocol for a systematic review and meta-analysis

**DOI:** 10.1097/MD.0000000000013953

**Published:** 2019-01-04

**Authors:** Yan Shen, Miao Liu, Jie He, Shu Wu, Yong-Lin Wan, Jun Ding, Xiao-Yan Cai, Xiao-Hong Fu

**Affiliations:** a Department of Medical Ultrasound; b Department of Pathology; c Department of Surgery, Gong Li Hospital, Pudong New Area, Shanghai, China.

**Keywords:** endoscopic thyroidectomy, meta-analysis, mortality rates, papillary thyroid microcarcinoma, ultrasound-guided percutaneous microwave ablation

## Abstract

**Background:**

The differences in treatment outcomes between ultrasound (US)-guided percutaneous microwave ablation (PMWA) and endoscopic thyroidectomy for patients with papillary thyroid microcarcinoma (PTMC) remains unknown.

**Methods:**

An electronic search will be performed for randomized controlled trials or observational studies that reported recurrence and mortality rates in PTMC patients with US-guided PMWA or endoscopic thyroidectomy. Hazard ratios with their 95% confidence intervals will be calculated using fixed- or random-effects models according to heterogeneity.

**Results:**

This study will present the differences in treatment outcomes between US-guided PMWA and endoscopic thyroidectomy for patients with PTMC by pooling the results of individual studies.

**Conclusion:**

The results of this study would provide vigorous evidence in this issue and offer the guidance to both clinical decision-making and future research.

**Ethics and dissemination:**

Ethical approval is not applicable for this study.

**PROSPERO registration number:**

CRD42018112320.

## 1 Introduction

Papillary thyroid carcinoma is the most common subtype (about 85%) in thyroid carcinoma, and lesions being 10 mm or less in largest dimension was defined as papillary thyroid microcarcinoma (PTMC).^[1]^ The incidence of PTMC has considerably increased owing to widespread screening and the technical improvements in thyroid ultrasonography and fine-needle aspiration biopsy.^[2–4]^ The American Thyroid Association guidelines recommended active surveillance for PTMC as the reported disease-specific mortality rate being less than 1%.^[5]^ However, for patients who had lymph node or distant metastases that might not accept active surveillance, surgery especially endoscopic thyroidectomy could be one of the appropriate treatments.^[6]^ The endoscopic thyroidectomy could also truly minimize the invasion and leave the scars concealed. Recently, microwave ablation has been proved to be a safe and effective technology for liver carcinoma and renal cancer, which could be an alternative to surgery.^[7]^ Microwave ablation has many advantages, including higher intratumoral temperature, ability to ablate lager volumes, as well as faster ablation speed.^[4]^ It could be preferable for patients without cervical metastatic lymph nodes, and are not willing to undergo surgery or surveillance, since it is also less-invasive choice. However, the comparison between these 2 therapeutic methods remains unclear. Therefore, this study is designed to do a systematic review and meta-analysis for investigating the differences in treatment outcomes between ultrasound (US)-guided percutaneous microwave ablation (PMWA) and endoscopic thyroidectomy for patients with PTMC.

## 2 Methods

### 2.1 Data sources and searches

The present study that is registered in PROSPERO (registration number: CRD42018112320) will be performed according to the standards of the Cochrane Handbook and the PRISMA Statement for Reporting Systemic Reviews.^[8,9]^ Ethical approval is not necessary because this is a systematic review and meta-analysis, which will not involve any subject directly. We will electronically search the databases of MEDLINE, Embase, and The Cochrane Library to identify all potentially eligible studies using the following searching strategy: “microcarcinoma” OR “papillary microcarcinoma” OR “small papillary carcinoma” OR “micropapillary carcinoma” OR “incidental carcinoma” OR “thyroid incidentaloma” OR “thyroid cancer” in combination with “thermal ablation” OR “microwave” OR “microwave ablation” OR “percutaneous microwave ablation” OR “ultrasound-guided percutaneous microwave ablation” OR “laser ablation” OR “radiofrequency ablation”. Moreover, unpublished data will be sought from the ClinicalTrials.gov website, and the references of identified records will be manually scrutinized to find any potentially eligible articles.

### 2.2 Study selection and outcomes

For study selection, the following inclusion criteria will be used:

(1) studies design should be limited to RCTs or observational studies that reported recurrence and mortality rates in PTMC patients with US-guided PMWA versus endoscopic thyroidectomy;
(2) only high-quality nationwide or health insurance database studies presenting adjusted or matched results will be eligible;
(3) only studies with the longest research period could be involved when same data sources were applied in different studies.

Two reviewers will evaluate all study titles and abstracts; thereafter full paper should be retrieved and assessed the relevant possibility according to the inclusion. To reduce bias, 2 reviewers should be blinded to journal, authors’ names, and year of publication of the papers. A third author should be consulted when any uncertainties and discrepancies appear. The primary outcome is a composite of recurrence and mortality rates. The secondary outcomes are operation time, postoperative hospitalization time, and postoperative complications reported in the study.

### 2.3 Data extraction, quality evaluation, and bias assessment

All data will be extracted independently by 2 reviewers using a priori designed form, including study characteristics, clinical characteristics, study duration, recurrence, and mortality rates. Interested outcomes which were not reported in the publications would be extracted from the ClinicalTrials.gov Website. Disagreements will be resolved by consensus after discussion or by consulting a third author. The methodological quality of the included RCTs will be evaluated using the Cochrane Collaboration's risk of bias tool.^[10]^ The methodological quality of the included observational studies will be assigned to the following domains:

(1) use of matched or adjusted method to handle selection bias;
(2) possibility for residual confounding;
(3) the use of methods to deal with time-varying covariates and information censoring; and
(4) detailed reporting of baseline characteristics and outcome measures.

Visual inspection of funnel plots will be used to explore the potential publication bias.^[11]^

### 2.4 Data analysis

Hazard ratios (HRs) and their 95% confidence intervals (CIs) will be calculated using fixed- and random-effects models. For the included randomized controlled trials (RCTs), HRs, and 95% CIs will be calculated, and for included observational studies, adjusted HR and 95% CIs will be pooled by using the METAN command for random-effects models in the statistical software package (STATA, version 12.0, Stata Corporation, College Station, TX). Heterogeneity will be evaluated using the *I*^2^ test.^[12]^ Subgroup analyses will be conducted for the study types (RCTs and database studies). Meta-regression analysis will be conducted to test the demographic characteristics of the involved studies. Sensitivity analyses will be performed to assess the robustness of results by sequentially eliminating individual studies. Potential publication bias will be assessed by visually inspecting funnel plots. *P* value of < .05 is considered as a statistically significant difference.

## 3 Discussion

With the increasing incidence of PTMC in recent decades, surgery has been recommended as the first-line treatment for the disease.^[7]^ However, conventional thyroidectomy could bring a large part of the thyroid away, which might inevitably cause hypothyroidism after surgery. Procedures such as endoscopic thyroidectomy and US-guided PMWA would not damage normal thyroid tissues and not cause postoperative hypothyroidism. Moreover, conventional thyroidectomy could leave a scar on patients within a highly visible area,^[13]^ and endoscopic thyroidectomy, as well as US-guided PMWA, as alternatives to surgery, could avoid this problem. It is well known that US-guided PMWA has many advantages, including higher intratumoral temperature, ability to ablate lager volumes, as well as faster ablation speed.^[4]^ Baek et al^[14]^ compared US-guided PMWA and conventional thyroidectomy in benign thyroid nodules treatment, and found that US-guided PMWA was characterized by a definite therapeutic success rate, good cosmetic effect, slight injury, and rapid recovery. Yue et al^[15]^ investigated the effectiveness and safety of US-guided PMWA in the treatment of benign thyroid nodules. The mean percent decrease in the volume of thyroid nodules was about 65% for 477 patients. All the above studies proved the effectiveness and practicability of PMWA in the PTMC treatment. However, little was known about the comparison between US-guided PMWA and endoscopic thyroidectomy. Therefore, we will conduct this systematic review and meta-analysis of published studies to investigate the differences in treatment outcomes between US-guided PMWA and endoscopic thyroidectomy for patients with PTMC. The findings of this study will make the understanding on the advantages as well as disadvantages of both procedures in PTMC, helping to make the decision on which procedure should be chosen with low recurrence and mortality rates.

## 4 Conclusions

The results of this study would provide vigorous evidence in this issue and offer guidance to both clinical decision-making and future research.

## Author contributions

Xiao-Hong Fu and Xiao-Yan Cai conceived the idea and design for this systematic review. Yan Shen and Miao Liu developed the methodology for the systematic review protocol. The contents of this manuscript were drafted by Yan Shen with input from all members of the authorship team. The manuscript was reviewed by Xiao-Hong Fu, Xiao-Yan Cai, Jie He, Shu Wu, Yong-Lin Wan, and Jun Ding for important intellectual content. All authors read and approved the final manuscript.

**Conceptualization:** Yan Shen, Miao Liu, Jie He, Shu Wu, Yong-Lin Wan, Jun Ding, Xiao-Yan Cai, Xiao-Hong Fu.
